# Effect of Chitosan’s Degree of Deacetylation on the Performance of PES Membrane Infused with Chitosan during AMD Treatment

**DOI:** 10.3390/membranes10030052

**Published:** 2020-03-24

**Authors:** Machodi Mathaba, Michael Olawale Daramola

**Affiliations:** 1 School of Chemical and Metallurgical Engineering, Faculty of Engineering and the Built Environment, University of the Witwatersrand, Private Bag X3, Wits 2050, Johannesburg, South Africa; 1259086@students.wits.ac.za; 2 Department of Chemical Engineering, Faculty of Engineering, Built Environment and Information Technology, University of Pretoria, Private bag X20 Hatfield, Pretoria 0028, South Africa

**Keywords:** polyethersulphone membrane, chitosan, degree of deacetylation, hydrophilicity, acid mine drainage

## Abstract

Acid mine drainage is an environmental problem associated with mining operations and activities. Its treatment is essential to achieving environmental sustainability. In this study, a polyethersulphone (PES) membrane infused with chitosan is proposed as a point-of-use material for treating acid mine drainage (AMD). The composite material explored the synergetic effect between chitosan and polymer, particularly considering the effect of the degree of deacetylation (DD) of chitosan on the performance of membrane. Chitosan was produced from chitin under various synthesis process conditions and infused within polyethersulphone membrane. The results obtained show that chitosan with the highest degree of deacetylation was achieved with a temperature of 100 °C and NaOH concentration of 40 wt%. Increasing the temperature above 100 °C started degrading already formed or exposed amine groups, thus, reducing the DD of the chitosan sample. The contact angle and porosity analysis indicated that the hydrophilic nature of the membrane was enhanced with increasing DD of the chitosan. The performance of the membranes was conducted on a Dead-end filtration cell using synthetic acid mine drainage. The results showed that the flux and rejection of the membrane was enhanced with increasing degree of deacetylation. PES 5 and PES 1 were blended with chitosan having the highest (95.97%) and lowest (33.93%) degree of deacetylation, respectively. PES 5 reported pure water flux of 123 L/m^2^·h and PES 1 was recorded as 104 L/m^2^·h. Similarly, the rejection of the membrane was improved with increasing chitosan’s degree of deacetylation. PES 5 had higher rejection and PES 1 had the least rejection. Maximum rejection for the contaminants was determined as 98.05, 97.39, 96.25, 95.24 and 80.34% for Mn^2+^, Fe^2+^, Mg^2+^ and Ca^2+^ and SO_4_^2−^, respectively. The results obtained show that chitosan’s degree of deacetylation has a positive effect on the performance of polyethersulphone membrane during the treatment of acid mine drainage.

## 1. Introduction

Membrane technology has gained significant attention for application in wastewater treatment due to high quality water requirements and environmental concerns. Membrane technology has proven to be a promising alternative to conventional processes due to its high separation efficiency and inexpensiveness [1]. However, during operation, membrane fouling restricts full performance of the membrane due to accumulation and deposition of contaminants on the membrane surface and within the pores. Numerous studies reviewed by [2,3] deduced that membrane hydrophilicity is directly related to membrane fouling. Therefore, hydrophilic modification of membranes during wastewater treatment is essential in membrane science and technology [4]. Natural or synthetic polymeric materials have attracted vast attention to be employed as membrane materials. Polymeric materials are attractive due to their non-toxic, and biodegradable properties and they cost effective [5]. Polyethersulphone (PES) has emerged as one of the most important polymeric material due to its high thermal and chemical resistance and mechanical stability [6]. The main disadvantage characterizing PES and PES based membranes is related to its hydrophobic character generated by the sulfonyl group linking the two phenylene rings [7]. Several alterations such as chemical, physical and surface modification and blending have been research to improve the hydrophilic property of PES membranes [8]. The ultimate aim of membrane modification with hydrophilic agents is to localize the hydrophilic material on the membrane surface and within the pores to positively influence membrane permselectivity and reduce fouling [4].

This study opted to blend hydrophilic chitosan within the PES membrane matrix to not only modify the membrane surface but also inside the pores. The synthesized membrane was performance was tested during the treated of acid mine drainage. Chitosan is obtained from chitin, which is a higher molecular weight linear polymer and is the second most abundant natural fiber after cellulose. Chitin is found in many different living organisms such as shellfish, crabs, insects, crustacean shells and arthropods [9]. Chitosan is obtained by partial N-deacetylation of chitin and it contains two free hydroxyl functional groups and one primary amino [10]. The large number of amino (–NH_2_) and hydroxyl (–OH) groups, which can act as contaminate binding sites, makes chitosan attractive, as it will improve both the membrane’s hydrophilic property and permselectivity. Under acidic medium, the amino group gets protonated and attract anions and repel cations through electrostatic repulsion [11]. Microporous chitosan/polyethylene glycol mixed matrix membrane was tested by Reiad et al. [12] for removal of iron and manganese from aqueous solution. The study showed improved iron and manganese rejection by the blend membrane and the authors concluded that its reusable after successful desorption of the rejected metal ions. Boricha and Murthy [13] synthesized and compared PES membrane coated chitosan and acrylonitrile butadiene styrene. It was found that increased chitosan content had a positive effect on the amorphous nature of the membrane. Permeate flux is directly related to amorphous nature of membranes.

Chitosan is obtained from chitin through a deacetylation process, that is treating chitin with a strong alkaline solution. The most important parameter characterizing chitosan sample is the degree of deacetylation [14]. Chitosan which is a principal derivative of chitin refers to partially or fully deacetylated chitin, which means the degree of acetylation is around or lower than 50%. This also means the degree of deacetylation is around or higher than 50%. Degree of deacetylation influences the physical, biological and chemical properties of the synthesized chitosan. Degree of deacetylation determines the free amino groups exposed due to the removal of the acetyl groups from the molecular chain of chitin, hence the name deacetylation. It is a parameter used to differentiate between chitin and chitosan [15]. Deacetylation process involves the removal of acetyl group from the molecular chain of chitin, leaving behind a complete amino group (–NH_2_) and chitosan versatility depends mainly on this high degree of chemical reactive amino groups. Chitosan with different chemical structures can be synthesized by manipulating reaction time, synthesis temperature and strength of the alkaline solution utilized during the deacetylation process. When chitosan is used to modify polymeric membranes for metal ion removal from solution, it is expected that a high number of available amino groups on the chitosan structure should translate into more effective sorption capacity. However, the influence of chitosan’s degree of deacetylation on the effectiveness of metal ion binding during AMD treatment is non-existent. The study first synthesizes chitosan with different degree of deacetylation by manipulating temperature and the strength of the alkaline solution. Then, chitosan with different degree of deacetylation was blended with PES membrane to evaluate the effect during acid mine drainage (AMD) treatment.

## 2. Experimental Setup 

### 2.1. Materials and Chemicals

Chitosan used in this study was synthesized from chitin which was obtained by processing seashells collected from Durban South Beach, Rutherford in KwaZulu Natal, South Africa. Chemicals such as Solvent Dimethyl Sulfoxide (DMSO), polyethersulphone (PES) granules (3mm), piperazine (PIP), trimesoyl chloride (TMC), triethylamine (TEA), acetone (C_3_H_6_O), sodium hydroxide (NaOH), hexane (C_6_H_14_), ethanol (C_2_H_6_O), sulphuric (H_2_SO_4_) and hydrochloric (HCl) acids and metal sulphates salts were obtained from Sigma-Aldrich (Pty), Johannesburg, South Africa. The chemicals were analytical grade; therefore, they were used without purification. Deionized water was prepared in-house by-passing tap water through Ion exchange polymer resins. The water had pH of 6.89 and conductivity of 0.19 mS/cm.

### 2.2. Production of Chitosan from Chitin

The seashells were washed and dried in an oven at 120 °C for 1 hour before crushing and milling into fine powder (chitin). The following steps were carried out chronologically:

(i) Deproteinization: The crushed and milled seashells (chitin) were treated with an alkaline NaOH (6 w/w%) solution in a 500 mL Erlenmeyer flask at 60 °C. The concoction was stirred on a heating plate fitted with a magnetic stirrer for 2 h. After 2 h of stirring, the chitin was separated from the solution by decanting the alkaline solution. The collected chitin was rinsed with deionized water until the pH was measured neutral.
(ii) Demineralization: After deproteinization, the resulting chitin was mixed for 2 h with 6% HCl solution in a 500 mL Erlenmeyer flask at 60 °C. After 2 h of mixing on heating plate equipped with a magnetic stirrer, the demineralized chitin was separated from the acidic solution by decanting the supernatant solution. The demineralized chitin was then washed with deionized water until neutral pH.
(iii) Deacetylation: The deproteinized and demineralized chitin was treated with various NaOH concentration (20, 40 and 60 wt%) and temperature (80, 100 and 120 °C) to manipulate the degree of deacetylation (DD) of chitosan. Nine chitosan samples were synthesized and stored inside airtight containers. The solid to liquid ratio for all processes was set at 1:20. Nine chitosan samples were obtained and Table 1 shows synthesis process conditions and corresponding chitosan sample No.

### 2.3. Fabrication of PES and Modified PES Membranes

PES granules were dissolved in Dimethyl Sulfoxide at room temperature measured at 26.8 °C on a magnetic stirrer. Once the PES granules dissolved, chitosan was added to the mix and was left for 24 h to obtain a homogenous gel. Before casting, the casting solution was left at ambient conditions to remove any air bubbles. The gel was cast at 250 µm thickness using a casting knife on a glass plate. The membrane was kept inside deionized water to allow for complete desorption of the solvent from the membrane sheet. The membranes were placed in oven at 60 °C to allow evaporation of any trapped water and/or solvent from the membrane for 15 min. 

### 2.4. Characterization of Chitosan and Membranes

Fourier Transform Infrared (FTIR) spectroscopy (Bruker Tensor 27 Spectrometer from LightMachinery Inc, Ottawa, Canada) was used to confirm success in chitosan synthesis from chitin and identifying chemical groups on the membrane surface. FTIR used a DTGS KBr dector and KBr beam splitter, set in a wavelength between 500 to 4500 cm^−1^ at an optical velocity of 0.6329. Thermogravimetric analysis was employed to determine the thermal stability of the PES membranes infused with chitosan samples having various degree of deacetylation. Thermal stability of the membranes was performed by heating the samples to 700 °C in air and the temperature. The TGA was performed using a TA SDT Q600 (TA Instruments, DE, USA) The wettability of the membranes was measured water drop using Dataphysics Optical contact angle analyzer (OCA 15 EC GOP from DataPhysics Instruments, Filderstadt, Germany) to quantify the hydrophilic property of the membranes. Ten random measurement were taken at different places on the membrane surface and the average value was utilized. The membranes bulk porosity was estimated gravimetrically. 2-propanol was used as a solvent to investigate the wettability of the membranes. Pieces of membranes were cut and placed inside the solvent for 24 h at room temperature. After 24 h, the pieces of the membranes were removed and placed between two filter papers to remove excess solvent on the surface. The pieces were weighed to obtain wet weight (W_w_). Subsequently, the wet membranes were heated in an oven for 2 h at 50 °C. The dried pieces were weighed to obtained dry weight (W_d_). The membranes bulk porosity was obtained using Equation (1):

(1) $$\mathrm{Porosity}\;\left(\%\right)=\frac{W_{w}-W_{d}}{A\times l\times d_{b}}\times 100$$

where, l is the average thickness of the membranes measured using a digital Micrometer, A is the membrane effective areas, $d_{b}$ is 2-propanol density (0.786 g/cm^3^).

Chitosan was characterized using FTIR. Although there are various techniques for chitosan characterization, infrared spectroscopy is the most discussed due to its simplicity [16]. As such, FTIR was employed to characterize chitosan and to determine its degree of deacetylation of the chitosan samples. Absorption band rations such as A_1655_/A_3450_, A_1560_/A_897,_ A_1320_/A_3450,_ A_1655_/A_2875_ and A_1655_/A_3450_ have been previously used to determine the DD of chitosan samples [17]. However, the absorption band ration of A_1320_/A_1420_ have proven to shows superior agreement between the absolute and estimated DD values (Habiba et al., 2017). The DD of the chitosan samples was determined using the following Equations (2) and (3) [18,19]:

(2) $$\mathrm{DA}\%=13.9(\frac{A1320}{A1420})-12.20$$

DD% = 1 − DA% (3)

DD% is the degree of deacetylation. Duplicate chitosan samples were prepared, and average values were taken. DA% is percentage degree of acetylation, and Percentage yield was determined to understand the efficiency of the synthesis process and conditions in terms of chitosan quantity which was obtained. Equation 4 was used to determine the percentage yield:

(4) $$\%\mathrm{Yield}=\frac{\mathrm{total}\;\mathrm{grams}\;\mathrm{of}\;\mathrm{products}\left(\mathrm{chitin}\right)}{\mathrm{total}\;\mathrm{grams}\;\mathrm{of}\;\mathrm{reactants}\left(\mathrm{chitosan}\right)}\times 100\%$$

### 2.5. Performance Evaluation of Fabricated Membranes Using Synthetic AMD

Synthetic AMD was used as feed solution in these experiments [18]. Synthetic AMD was used as feed to avoid competition of desired contaminates with undesired species present in real industrial AMD. Suitable amount of metal sulphate salts (Table 2) were dissolved and agitated at 200 rpm for 30 min in 1000 mL of deionized water to ensure complete dissolution and 0.1 M sulphuric acid was used to adjust the pH to 3.2 using 0.1 M sulphuric acid. To ensure consistent quality of the synthetic AMD, the AMD solution was prepared and used on the same day without storage. 

Membrane performance was conducted on a laboratory-scale Dead-end filtration setup consisting of a holding cell (300 mL volume) and effective filtration area of 14.6 cm^2^. Nitrogen gas was used to achieve the desired pressure. The membranes were pre-pressed and compacted with deionized water to ensure complete immersion of water. Pure water flux (J, L/m^2^·h) of the membranes was determined by permeating deionized water through the membrane at ambient temperature to obtain the original flux of the membranes. Pure water flux (J, L/m^2^·h) was determined by direct measurement of membrane permeate volume using the following Equation (5):

(5) $$J=\frac{V}{\mathrm{At}}$$

where V (Liters) is the volume of permeated water, A (m^2^) is the effective membrane area and t (h) is the filtration time. To minimize errors, water flux and rejection experiments were carried out three times and average values were reported.

The filtrates were collected and analyzed for metal content using Atomic Absorption Spectroscopy (Thermo scientific ICE 3000 from ThermoFischer Scientific, Waltham, USA). Table 3 depicts operating conditions of the AAS. Sulphates were determined using Uv-vis spectrophotomer (PG instruments T60 from Alma Park, Leicestershire, United Kingdom) following the Environmental Protection Agency method (EPA method 3754). Samples for sulphates analysis were conditioned with a conditioning solution prepares as follows: 100 mL 95% ethanol was mixed with 30 mL of HCl and 75 g NaCl in a 500 mL flask. Then glycerol was added to the mixture. For sulphates analysis, 1 mL of the filtrates and 5 mL of the conditioning solution were transferred mixed on a magnetic stirrer. Thereafter, a spoonful of BaCl_2_ was added to the mix and stirring continued for an additional 5 min. After stirring, the solution was placed into a cuvette for 4 min at 30 s interval to obtain the turbidity of the solution. A calibration curve was prepared by appropriate dilution of 100 ppm Na_2_SO_4_ bulk solution. 

Rejection was determined with the following Equation (6):

(6) $$R\left(\%\right)=\frac{C_{\mathrm{feed}}-C_{\mathrm{permeate}}}{C_{\mathrm{feed}}}\times 100\%$$

where R is the percentage rejection and $C_{\mathrm{feed}}$ and $C_{\mathrm{permeate}}$ (mg/L) are feed and permeate concentrations, respectively.

## 3. Results and Discussion

### 3.1. Membrane and Chitosan Characterization

#### 3.1.1. Fourier Transform Infrared Analysis of the Chitosan and Membranes

**Figure 1:**
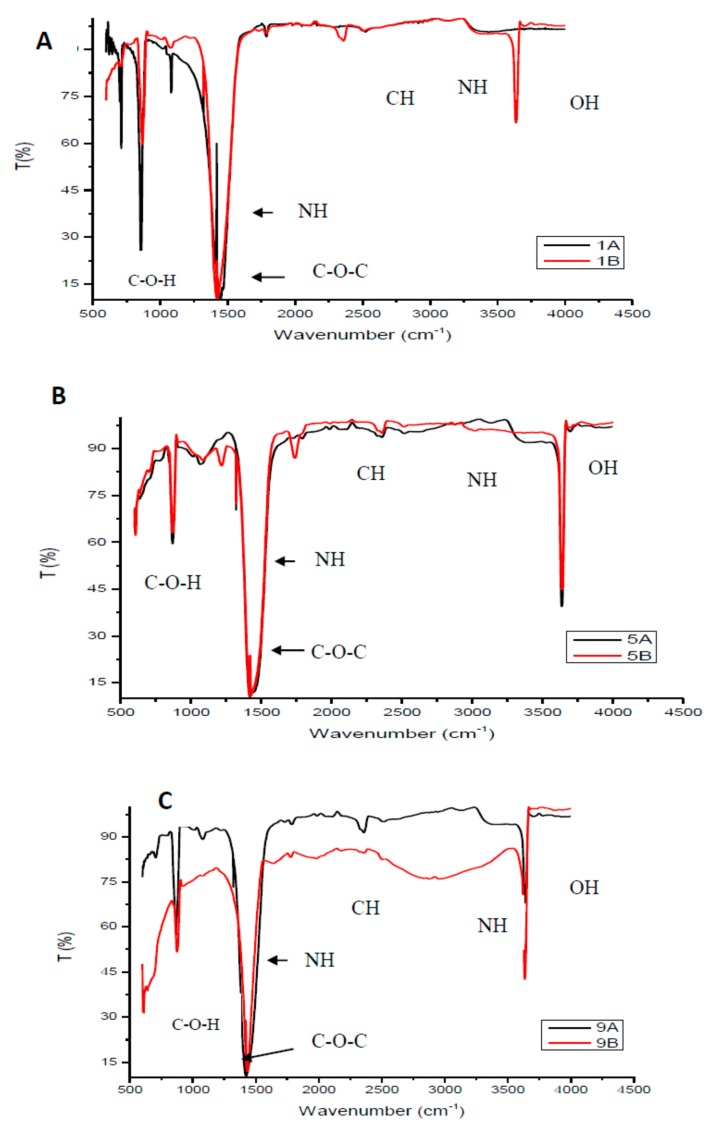
Fourier Transform Infrared spectra of selected chitosan (**A**) Samples 1A and 1B, (**B**) Samples 5A and 5B, (**c**) Samples 9A and 9B.

Figure 1 shows FTIR spectra of the selected chitosan samples (Samples 1, 5 and 9). Duplicate samples were prepared to confirm accuracy and repeatability of the synthesis process. The FTIR spectra of the chitosan samples exhibited characteristic amino peaks at around 3300 to 3500 cm^−1^ overlapping with the N–H stretching with a minimum intensity of the amine functional group. The observed bending vibration at around 1450 to 1480 cm^−1^ was assigned to the N–H bending vibration of the R–NH_2_ functional group which indicate an increased degree of deacetylation. The typical alcohol peaks (OH and COH) are observed to occur at around 3500 cm^−1^ and just before 1000 cm^−1^. According to the study conducted by Marei et al. [20] the absorption peaks observed at around 1080 cm^−1^ are due to the C–O–C stretching. The peaks observed between 2400 and 2800 cm^−1^ indicate the alkaline C–H vibration of CH_2_ [21]. Studies in literature revealed FTIR spectroscopies of chitosan samples having similar characteristic peaks obtained in this study [22]. This indicates successful chitosan synthesis from chitin. The large presence of various functional groups available on the chitosan structure formed basis of why chitosan was selected to modify the PES membrane. The protonation of available NH and NH_2_ functional groups under acidic medium will favor electrostatic repulsion and attraction of cations and anions, respectively. 

In order to easily identify the synthesized membranes, Table 4, presents the coded names of the synthesized membranes which corresponds with the chitosan sample number that was used to modify that membrane. 

**Figure 2:**
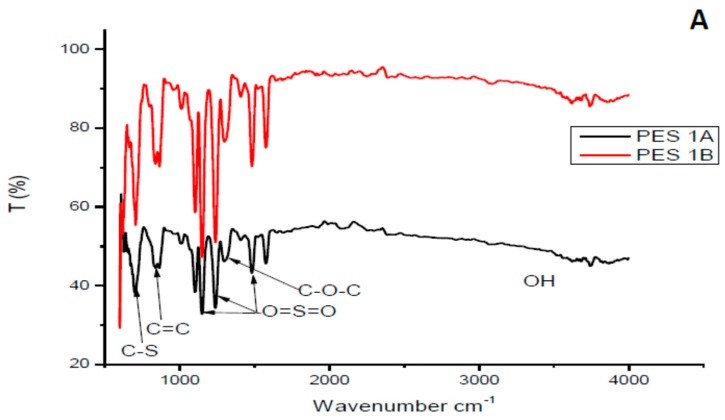

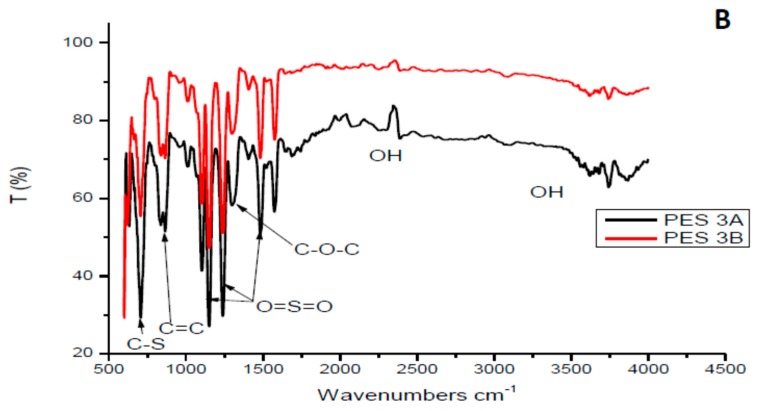
FTIR spectra of selected synthesized PES/chitosan membranes chitosan (**A**) Samples 1A and 1B, (**B**) Samples 3A and 3B.

Figure 2 depicts FTIR spectra of both PES/chitosan (PES 1 and PES 3). The chitosan samples had a different degree of deacetylation. Looking at the FITR spectra, the effect of chitosan’s degree of deacetylation could not be verified. The spectra of the membranes were attributed to the properties of basic PES structure as no significant different between PES and PES/chitosan membranes could be observed. FTIR spectra of the synthesized chitosan samples did not show a significant difference. Similar observations were made by Chen et al. [23]. Additionally, Boricha and Murthy [14] observed no structural differences on the surface of PES membranes modified with different chitosan concentration. In this study, the FTIR spectra of membranes modified with 0.75 wt% chitosan had similar characteristics with those prepared having chitosan with various degree of deacetylation. The C-stretching and C=C stretching on the aromatic rings were identified with peaks at 620 and 880 cm^−1^, respectively. The PES characteristic sulfonyl group was confirmed with peaks at 1150 cm^−1^, 1239 cm^−1^ and 1483 cm^−1^ The aromatic ether (C–O–C) group was assigned to the peak at 1244 cm^−1^. Moreover, FTIR spectra confirm that chitosan’s degree of deacetylation does not have significant impact on the surface structure of the membranes. 

#### 3.1.2. Chitosan Yield

Percentage yield was determined to understand the efficiency of the synthesis process and conditions in terms of chitosan quantity, which was obtained using Equation (4). Table 5 presents the overall yield for all nine chitosan samples.

Crushed and milled chitin was in flakes structure and the intensity of the dark brown color shifted from light to dark brown after the demineralization, deproteinization and deacetylation process. The percentage yield was low for all nine samples and this is attributed to the nature of the synthesis process. Removal of minerals and proteins from chitin via demineralization and deproteinization accounted for this huge percentage mass loss. Percentage yield of chitosan from chitin decreased as the concentration of NaOH and temperature increased, respectively. Higher temperature and NaOH concentration showed to have high capabilities to remove proteins from the chitin structure. Similar observations were made by Soon et al. [24] and Srinivasan et al. [21]. Srinivasan et al. [21] reported yield of 35% using 50% NaOH concentration at 90 °C for 50 min as deacetylation conditions. Chitosan yield in the range of 4.77–5.43% was realized by Soon et al. [24] using deacetylation conditions of 50% NaOH at 90 °C for 30 h. These conditions are closely similar to deacetylation conditions used to synthesis sample 2 and 5 in this study, respectively. Sample 2 and Sample 5 displayed yields of 25.3 and 19.8%, respectively. The higher yield reported by Srinivasan et al. [21] as comparable to sample 2, and the results for sample 5 could be attributed to the low synthesis time of 50 min as compared to 6 h used in this study. Samples 6, 7, 8 and 9 had even lower yields and this could be attributed to either increased synthesis temperature and/or NaOH concentration.

#### 3.1.3. Degree of Deacetylation of Chitosan

The degree of deacetylation (DD) of chitosan determines the amount of acetyl groups that have been removed from the chitosan structure, leaving behind free amino groups on the polysaccharide. The greater the DD, the more available amine groups are exposed as potential binding sites for contaminants. Studies have concluded that temperature, reaction time and NaOH concentration have a significant effect on the DD of the synthesized chitosan. Deacetylation process is achieved by treating chitin with concentrated NaOH or KOH (40 to 50%) usually at around 100 °C for several h [25]. Palpandi et al. [26] synthesized chitosan by treating chitin with 40% NaOH solution at 110 °C for 6 h, Kumari et al. [22] synthesized chitosan from fish scales, shrimp and crab shells using 40% KOH at various temperatures for 6 h, and Hussain et al. [14] synthesized chitosan by treating chitin with 40% NaOH at 80 °C for 4 and 8 h. In the present case, the reaction time was kept constant at 6 h and temperature (80 °C, 100 °C and 120 °C) and NaOH (20%, 40% and 60%) concentration was varied to the synthesis chitosan samples having different DD.

Table 6 shows the determined DD of chitosan samples synthesized under different deacetylation conditions. The general trend observed was that DD increased from 33.9 to 73.05% when the NaOH concentration was increased from 20% to 40% when the synthesis temperature was kept constant at 80 °C, respectively. Similar observations were made when synthesis temperature was increased to 100 °C and 120 °C for the same NaOH concentration (from 20 to 40%, respectively). This is due to the higher strength of the NaOH solution, which promotes the degradation of the acetyl group and formation or exposure of more amine groups, thus increasing the DD of the sample. Increasing NaOH concentration to more than 40% induced reduction in DD of the chitosan. DD was reported as 73.05% when the NaOH was 40% at 80 °C but increasing NaOH concentration to 60% at the same temperature reduced the DD to 60.82%. This indicated that NaOH concentration of greater than 40% encourages the degradation of the chitosan sample. Similar observations were made in the case of temperature in isolation. The DD increased from 73.05 to 95.97% when the temperature was increased from 80 °C to 100 °C and NaOH concentration was kept constant at 40%, respectively. This is attributed to the greater kinetic energy induced by high higher temperatures which causes degradation of the acetyl groups as more reactions occurs. When more acetyl groups are degraded, more amine groups are exposed, thus increasing the DD of the chitosan. Increasing the temperature to 120 °C caused a decline in DD of the chitosan sample. As the temperature was increased to 120 °C, there was reduction in DD. Based on this behavior, it can be argued that at temperatures above 100 °C, the chitosan sample started to degrade and destroying the already formed or exposed amine groups, thus reducing the DD of the chitosan sample.

Kumari et al. [22] reported DD of 70%, 75% and 78% by extracting chitosan from fish, crab and shrimp shells, respectively, using 40% NaOH concentration at for 6 h at 90 °C. The deacetylation conditions used by Kumari et al. [22] were similar to the conditions used to synthesize sample 2, 6 and 8, with a slight difference in temperature. The obtained DD in this study were different from the DD obtained by Kumari et al. [22]. The DD obtained at 80, 100 and 120 °C was reported as 73.05, 95.97 and 90.17%, respectively. For both NaOH concentration and temperature in isolation, the DD increased up to a certain point and then decreased when the other is kept constant. At a temperature of 120 °C and NaOH concentration of 60 % the DDA is 85.55 %; if the temperature is decreased by a ratio of 1.5 to 80 °C, the DD decreases to 60.82%. In contrast, when NaOH concentration is decreased by a ratio of 1.5 from 60 to 40% at the same synthesis temperature, the DD increased to 90.15%. Thus, there is a decrease in the DD when the temperature is reduced and an increase in the DD when NaOH concentration is decreased by the same ratio. Thus, temperature is more significant than NaOH concentration. A higher DD of 95.97% was achieved with a temperature of 100 °C and NaOH concentration of 40%.

#### 3.1.4. Degree of Hydrophilicity (Contact Angle Measurement) and Porosity Analysis

**Figure 3:**
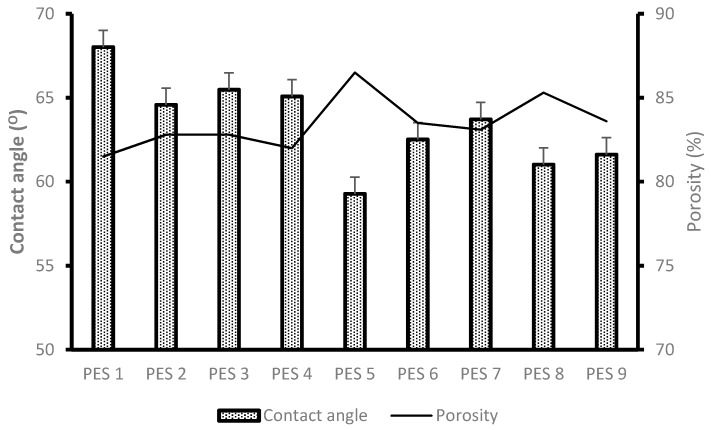
Contact angle and porosity results of PES/chitosan membranes.

The contact angle and porosity results for the synthesized PES/chitosan membranes are depicted in Figure 3. A relatively low contact angle indicates a hydrophilic nature of the membrane. It has been deduced that blending hydrophilic chitosan inside the PES membrane matrix will enhance the hydrophilic nature of the membrane. It is known that there is water transport through the membranes as a result of water molecules interaction with amide of the hydrophilic chitosan through hydrogen bonding. Therefore, a high degree of deacetylation is expected, which means more amine groups available on the chitosan molecule will enhance water transport through the membrane. PES 5 was blended with chitosan having the highest degree of deacetylation and both reported the lowest contact angle values of 59.28°. The same membrane reported the highest porosity due to enhanced hydrophilic natures as compared with other membranes. PES 1 was modified with chitosan having the lowest degree of deacetylation of 33.93% and it reported a relatively high contact angle values of 68.02°, indicating a more hydrophobic character. This was due to the limited number of functional groups available to facilitate water movement on the membrane surface and within. Based on the results depicted in Figure 3 the general trend is that high degree of deacetylation of chitosan enhances the degree of hydrophilicity and porosity of the membranes. 

#### 3.1.5. Thermal Stability of Membranes

**Figure 4:**
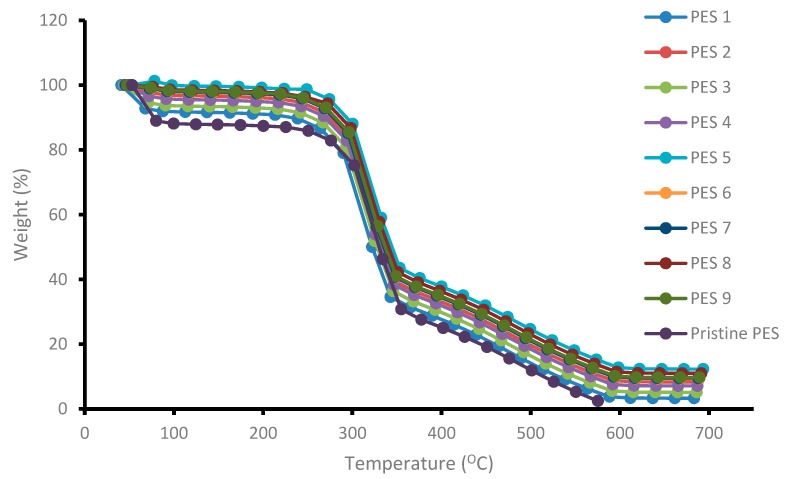
Thermal stability of the synthesized PES/chitosan membranes.

The thermal degradation and stability of the synthesized membranes blended with chitosan having various degree of deacetylation was evaluated and determined using thermogravimetric analyzer. The results are depicted in Figure 4. The general trend observed was that the thermal stability plot showed loss of mass of the membranes with increasing temperature. A three-step degradation process was observed, which were at 65 °C, 302 °C and 350 °C. The rapid degradation or decrease in weight percent from 0 to 100 °C was due to the evaporation of moisture and other gases present on the chitosan surface (First degradations step). Although PES membranes do not contain water molecules, FTIR spectra showed an OH functional group, which was attributed to some water molecules still trapped inside the membranes matrix even after drying. Addition of chitosan polymer inside the membranes matrix also contributed to the mass loss due to hydroxyl group evaporation present on the chitosan molecule. The second and third weight degradation steps could be attributed to the destruction of the D-glucosamine and N-glucosamine on the chitosan structure respectively. As can be observed in Figure 4, the addition of chitosan inside the membrane’s matrix improved the thermal stability of the membranes as the graph shifted to the right showing high temperature tolerance. A high degree of deacetylation signifies a higher presence of D-glucosamine and N-glucosamine than acetyl groups. The weight loss and corresponding temperature difference for the samples is attributed to the difference in degree of deacetylation of the chitosan samples used. PES 5, PES 8 and PES 9 were shown to be more thermally stable than the other membranes, and this could be attributed to the fact that the chitosan used to modify the membranes had higher degree of deacetylation of 95.97%, 90.17% and 85.55%, respectively. More heat was required to destroy the formed D-glucosamine and N-glucosamine. Figure 4 indicate that all the membranes were thermally stable at up to 302 °C, whereby a rapid membrane loss was observed. Another membrane loss was observed beyond 380 until up to ±585 °C. The high thermal stability could also be attributed to the long molecular chain and high molecular weight as well as the benzene rings connected by the sulfonyl group on the PES structure [27]. 

### 3.2. Membrane Performance Evaluation

**Figure 5:**
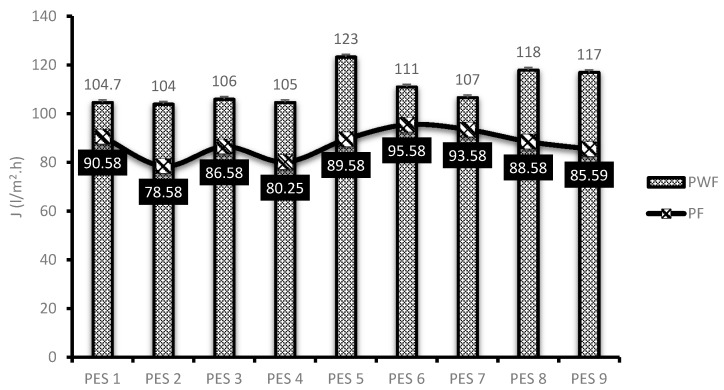
Membrane flux of PES/chitosan membranes.

Membrane performance evaluation was conducted as described in Section 2.5. Figure 5 depicts the original water flux (PWF) and flux of permeate AMD (PF) solutions for the nine synthesized PES/chitosan membranes. The membranes were pre-pressed with deionized water for 4 h to obtain a steady flux before commencing with actual flux determination. This was necessary to ensure complete immersion of water in the membranes. The highest PWF of PES/chitosan was measured as 123 L/m^2^·h for PES 5. The lowest was measured as 104 L/m^2^·h for PES 1. Observing the FTIR spectra of the membranes reported on Figure 2, they all had similar surface chemistry; however, the PWF results varied. This variation could be attributed to the difference in degree of deacetylation of chitosan samples (Table 4) used in the blend to synthesis the membranes. Membranes blended with chitosan having the highest degree of deacetylation reported high PWF and those with low degree of deacetylation reported low PWF. A high degree of deacetylation implies a large number of amines groups exposed after the deacetylation process. The high PWF with a high degree of deacetylation was attributed to the number of amines groups which favored sorption of water molecules inside the membrane matrix. Moreover, contact angle and porosity results presented in Figure 3 corroborated that hydrophilic nature of the membranes and porosity had a positive impact on the membranes PWF. Membranes with high degree of hydrophilicity and porosity reported high PWF.

**Figure 6:**
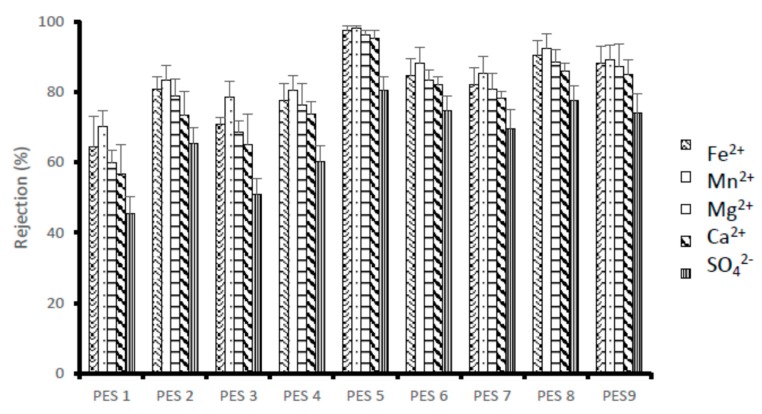
Rejection (%) of metal and sulphates ions using PES/chitosan membranes.

Figure 6 illustrate the rejection of ions by the synthesized PES/chitosan membranes using Dead-End filtration. AMD solution was passed through a Dead-End setup and permeate solutions were collected to determine the flux and analyzed for metal and sulphate ions content. Three runs were conducted, and the rejection was averaged. All the membranes had similar chitosan content loading of 0.75 wt%; however, the chitosan samples had different degree of deacetylation, which were obtained using different synthesis conditions (Table 4). A high degree of deacetylation signifies a large number of amine group exposed during the deacetylation process. It is expected that membrane rejection will increase with increasing chitosan’s degree of deacetylation due to the high number of amine groups playing important role during rejection. The general observed trend for all the contaminants was that, there is an increase in rejection with increasing degree of deacetylation. This observation is consistent with literature because increasing number of functional groups enhances membrane rejection capacity [28]. This is affirmed by PES 1, which was blended with chitosan having the lowest degree of deacetylation reporting low rejection for all metal and sulphate ions. Additionally, PES 5 blended with chitosan with the highest degree of deacetylation of 95.97% had the highest rejections. 

Without measuring the surface charge of the membranes, studies have shown that polymeric membranes are usually positively and negatively charged at lower and higher pH solutions, respectively [29]. pH of the feed used in this test was 3.2. Consequently, the higher rejection of cations (Mn^2+^, Fe^2+^, Mg^2+^ and Ca^2+^) as compared to anions (SO_4_^2−^) could be attributed to the strong repulsive forces dominance between the positively charged membranes and the metal ions. A high degree of deacetylation signifies more amine groups exposed during the deacetylation process, which can become potential binding sites. As the amine groups gets protonated, the repulsion and attraction forces will be stronger compared to the membranes having chitosan with low degree of deacetylation. PES 5 with chitosan having the highest degree of deacetylation (95.97%) reported maximum rejection of 98.05, 97.39, 96.25, 95.24 and 80.34% for Mn^2+^, Fe^2+^, Mg^2+^ and Ca^2+^ and SO_4_^2−^, respectively. PES 1 with chitosan having the lowest degree of deacetylation had poor rejection for Mn^2+^, Fe^2+^, Mg^2+^ and Ca^2+^ and SO_4_^2−^ at 70.15, 64.25, 59.80, 56.58 and 45.53%, respectively. The general observed rejection percentage trend is Mn^2+^ > Fe^2+^ > Mg^2+^ > Ca^2+^ >SO_4_^2−^, respectively. The higher rejection of Fe^2+^ in comparison to Mg^2+^ could be argued by what by Mthethwa [30] had observed. This could be attributed to the fact that Fe^2+^ tend to form stable chitosan-metal complex compared to Mg^2+^ and Mn^2+^.

## 4. Conclusions

This study sought to synthesis and evaluate the performance of PES membrane infused with chitosan for the treatment and acid mine drainage. First, chitosan was produced from chitin following different synthesis conditions. Secondly, the produced chitosan was blended within the PES membrane. Based on the obtained results, the following conclusions can be drawn:

1. Chitosan with the highest degree of deacetylation of 95.97% was reported with 40% NaOH concentration and temperature of 100 °C.
2. Percentage yield of chitosan from chitin decreased as the concentration of NaOH and temperature increased, respectively. Higher temperature and NaOH concentration showed to have high capabilities to remove proteins from the chitin structure, thus, the low yield was expected.
3. PES 5 was blended with chitosan having the highest degree of deacetylation of 95.975% and both reported the lowest contact angle values of 59.28°. Similarly, PES 1 was modified with chitosan having the lowest degree of deacetylation and showed to be hydrophobic.
4. Pure water flux of the membranes showed to be enhanced with increasing degree of deacetylation.
5. Similar behavior was observed for rejection investigations of the membranes. PES 5 reported high rejection and PES 1 had the least rejection. PES 5 with chitosan having the highest degree of deacetylation (95.97%) reported maximum rejection of 98.05, 97.39, 96.25, 95.24 and 80.34% for Mn^2+^, Fe^2+^, Mg^2+^ and Ca^2+^ and SO_4_^2−^, respectively.

## Figures and Tables

**Table 1:** Chitosan synthesis process conditions.

Chitosan Sample No.	Synthesis Process	Conditions
	Temperature( °C)	NaOH Conc (wt%)
1	80	20
2	80	40
3	80	60
4	100	20
5	100	40
6	100	60
7	120	20
8	120	40
9	120	60

**Table 2:** Composition of the synthetic Acid Mine Drainage.

Species	Concentration (mg/L) pH = 3.2	Salt Dissolved
Fe^2+^	933	FeSO_4_·7H_2_O
Ca^2+^	461	CaSO_4_·2H_2_O
Mg^2+^	345	MgSO_4_·7H_2_O
Mn^2+^	321	MnSO_4_·H_2_O
SO_4_^2−^	4556	Na_2_SO_4_

**Table 3:** Atomic Absorption Spectroscopy operating parameters.

Metal	Lamp Current	Wavelength	Flame Used
	(mA)	(nm)	
Al	10	309.27	Air/Acetylene/N_2_O
Ca	18	422.67	Air/Acetylene
Co	10	240.73	Air/Acetylene
Cu	4	324.75	Air/Acetylene
Fe	15	248.30	Air/Acetylene
Mg	18	285.21	Air/Acetylene
Mn	25	279.50	Air/Acetylene
Na	5	589.00	Air/Acetylene
Ni	5	232.00	Air/Acetylene

The instrument mode used was flame; the spectrometer used absorbance measurement mode with a bandpass of 0.5 nm and it was resampled three times; the fuel flow was at 4.2 L/min and the burner height was 11 mm. The nebulizer update was 4 s.

**Table 4:** Coded names of the synthesized membranes.

Chitosan Sample No.	%Degree of Deacetylation	PES/Chitosan Membranes Coded Names
1	33.93	PES 1
2	73.05	PES 2
3	60.82	PES 3
4	61.94	PES 4
5	95.97	PES 5
6	82.92	PES 6
7	79.92	PES 7
8	90.17	PES 8
9	85.55	PES 9

**Table 5:** Chitosan yield.

Chitosan Sample No.	%Yield	Standard Deviation
1	28.8	5.4
2	25.3	1.9
3	18.2	2.6
4	23.8	4.0
5	19.8	3.5
6	14.2	1.6
7	13.7	4.2
8	12.7	0.9
9	10.8	1.6

**Table 6:** Degree of deacetylation of chitosan under different synthesis conditions.

Sample No.	Experimental Conditions		DD%	Standard Deviation
	Temperature (°C)	NaOH Conc (wt%)		
1	80	20	33.93	1.41
2	80	40	73.05	5.20
3	80	60	60.82	1.67
4	100	20	61.94	0.10
5	100	40	95.97	1.49
6	100	60	82.92	2.65
7	120	20	79.92	3.59
8	120	40	90.17	0.82
9	120	60	85.55	5.11
